# Correlations between 4β-hydroxycholesterol and hepatic and intestinal CYP3A4: protein expression, microsomal ex vivo activity, and in vivo activity in patients with a wide body weight range

**DOI:** 10.1007/s00228-022-03336-9

**Published:** 2022-06-01

**Authors:** Kine Eide Kvitne, Kristine Hole, Veronica Krogstad, Birgit Malene Wollmann, Christine Wegler, Line K. Johnson, Jens K. Hertel, Per Artursson, Cecilia Karlsson, Shalini Andersson, Tommy B. Andersson, Rune Sandbu, Jøran Hjelmesæth, Eva Skovlund, Hege Christensen, Rasmus Jansson-Löfmark, Anders Åsberg, Espen Molden, Ida Robertsen

**Affiliations:** 1 Section for Pharmacology and Pharmaceutical Biosciences, Department of Pharmacy, University of Oslo, Blindern, P.O. Box 1068, 0316 Oslo, Norway; 2 Center for Psychopharmacology, Diakonhjemmet Hospital, Oslo, Norway; 3 Department of Life Sciences and Health, Oslo Metropolitan University, Oslo, Norway; 4 Department of Pharmacy, Uppsala University, Uppsala, Sweden; 5 DMPK, Research and Early Development, Cardiovascular, Renal and Metabolism (CVRM), AstraZeneca, BioPharmaceuticals R&D, Gothenburg, Sweden; 6 The Morbid Obesity Center, Vestfold Hospital Trust, Tønsberg, Norway; 7 Department of Pharmacy and Science for Life Laboratory, Uppsala University, Uppsala, Sweden; 8 Clinical Metabolism, Cardiovascular, Renal and Metabolism (CVRM), Late-Stage Development, AstraZeneca, BioPharmaceuticals R&D, Gothenburg, Sweden; 9 Department of Molecular and Clinical Medicine, Institute of Medicine, Sahlgrenska Academy, University of Gothenburg, Gothenburg, Sweden; 10 Oligonucleotide Discovery, Discovery Sciences, R&D, AstraZeneca, Gothenburg, Sweden; 11 Deparment of Surgery, Vestfold Hospital Trust, Tønsberg, Norway; 12 Department of Endocrinology, Morbid Obesity and Preventive Medicine, Institute of Clinical Medicine, University of Oslo, Oslo, Norway; 13 Department of Public Health and Nursing, Norwegian University of Science and Technology, NTNU, Trondheim, Norway; 14 Department of Transplant Medicine, Oslo University Hospital, Oslo, Norway

**Keywords:** 4β-Hydroxycholesterol, CYP3A4, Midazolam pharmacokinetics, Drug metabolism, Proteomics

## Abstract

**Purpose:**

Variability in cytochrome P450 3A4 (CYP3A4) metabolism is mainly caused by non-genetic factors, hence providing a need for accurate phenotype biomarkers. Although 4β-hydroxycholesterol (4βOHC) is a promising endogenous CYP3A4 biomarker, additional investigations are required to evaluate its ability to predict CYP3A4 activity. This study investigated the correlations between 4βOHC concentrations and hepatic and intestinal CYP3A4 protein expression and ex vivo microsomal activity in paired liver and jejunum samples, as well as in vivo CYP3A4 phenotyping (midazolam) in patients with a wide body weight range.

**Methods:**

The patients (*n* = 96; 78 with obesity and 18 normal or overweight individuals) were included from the COCKTAIL-study (NCT02386917). Plasma samples for analysis of 4βOHC and midazolam concentrations, and liver (*n* = 56) and jejunal (*n* = 38) biopsies were obtained. The biopsies for determination of CYP3A4 protein concentration and microsomal activity were obtained during gastric bypass or cholecystectomy. In vivo CYP3A4 phenotyping was performed using semi-simultaneous oral (1.5 mg) and intravenous (1.0 mg) midazolam.

**Results:**

4βOHC concentrations were positively correlated with hepatic microsomal CYP3A4 activity (*ρ* = 0.53, *p* < 0.001), and hepatic CYP3A4 concentrations (*ρ* = 0.30, *p* = 0.027), but not with intestinal CYP3A4 concentrations (*ρ* = 0.18, *p* = 0.28) or intestinal microsomal CYP3A4 activity (*ρ* = 0.15, *p* = 0.53). 4βOHC concentrations correlated weakly with midazolam absolute bioavailability (*ρ* =  − 0.23, *p* = 0.027) and apparent oral clearance (*ρ* = 0.28, *p* = 0.008), but not with systemic clearance (*ρ* =  − 0.03, *p* = 0.81).

**Conclusion:**

These findings suggest that 4βOHC concentrations reflect hepatic, but not intestinal, CYP3A4 activity. Further studies should investigate the potential value of 4βOHC as an endogenous biomarker for individual dose requirements of intravenously administered CYP3A4 substrate drugs.

**Trial registration:**

Clinical.Trials.gov identifier: NCT02386917.

**Supplementary information:**

The online version contains supplementary material available at 10.1007/s00228-022-03336-9.

## Introduction

The cytochrome P450 (CYP) 3A subfamily, consisting mainly of CYP3A4 and the polymorphic CYP3A5 [1], plays a significant role in the metabolism of 30–50% of clinically used drugs [2, 3]. Due to its abundant expression in both the liver and small intestine [4], CYP3A4 contributes significantly to the first-pass and systemic metabolism of substrate drugs. Hence, CYP3A4 is an important determinant for oral bioavailability and systemic clearance and thereby systemic drug exposure. There is substantial inter-individual variability in CYP3A4 activity, mainly due to environmental factors, disease state, and drug-drug interactions, leading to differences in dose requirements between patients. Conditions such as obesity and non-alcoholic fatty liver disease (NAFLD) are associated with a lower expression and activity of CYP3A4, and several studies suggest that body weight is inversely associated with CYP3A4 activity [5–10]. So far, genetic factors seem to be of limited importance for the substantial variability in CYP3A4 phenotype [11, 12]. Thus, non-genetic biomarkers are warranted to study individual variability in CYP3A4 activity in humans.

The current gold standard method to assess in vivo CYP3A4 activity is midazolam, a selective CYP3A4 phenotypic probe drug in vivo with a short half-life [13–16]. However, since midazolam is a medium-to-high extraction ratio drug, systemic clearance may be affected by changes in protein binding, hepatic blood flow, and intrinsic clearance (CL_int_), the latter representing metabolic capacity [17–19]. Previous studies have suggested that midazolam pharmacokinetics may be influenced by an increased hepatic blood flow in patients with obesity [20, 21]. Thus, in selected patient populations, the use of midazolam for CYP3A4 phenotyping may be challenging as it does not necessarily reflect CYP3A4 activity. 4β-hydroxycholesterol (4βOHC), a cholesterol metabolite mainly produced by CYP3A4, is proposed as a promising endogenous biomarker for CYP3A4 [22, 23]. There are practical advantages with an endogenous biomarker such as 4βOHC; it is non-invasive, and only a single blood sample is required, making it more convenient for measuring CYP3A4 activity than traditional pharmacokinetic studies with CYP3A4 probe drugs. To assess its suitability as a marker for CYP3A4 phenotype, several studies have investigated the correlation between plasma concentrations of 4βOHC and apparent oral clearance (CL/F) or systemic clearance of midazolam, but the results are conflicting [24–27]. The current literature suggests that 4βOHC concentrations reflect inter-individual variability in CYP3A4 activity and have mainly been suggested as a marker for CYP3A4 induction [28–30]. Still, the long elimination half-life may limit the ability to observe acute changes in CYP3A4 activity [31]. Also, the contribution of intestinal CYP3A4 in the formation of 4βOHC remains unclear [32]. The potential use of 4βOHC as a biomarker for individualized oral dosing of CYP3A4 substrates has thus been debated [33–35].

Additional investigations are required before 4βOHC can be accepted as a validated biomarker. As part of the primary endpoint in the COCKTAIL study [32], we evaluated 4βOHC as an endogenous biomarker for CYP3A4 activity by investigating the correlations between 4βOHC and different CYP3A4 metrics: (1) CYP3A4 expression and (2) ex vivo microsomal CYP3A4 activity in paired liver and jejunum biopsies and (3) absolute bioavailability, apparent oral clearance, and systemic clearance of midazolam in vivo.

## Methods

### Patients

In total, 96 patients from the open-label, three-armed COCKTAIL-study (NCT02386917) were included in the present analysis [36, 37]. The study population included 78 patients with severe obesity (BMI -> 40 or 35–40 kg/m^2^ combined with at least one obesity-related comorbidity) scheduled for weight loss treatment with Roux-en-Y-gastric bypass (RYGB) (*n* = 38) or non-surgical calorie restriction (*n* = 40), and 18 mainly normal to overweight individuals scheduled for cholecystectomy (BMI 18.5–30 kg/m^2^). The patients with severe obesity were subjected to a 3-week low-energy diet (< 1200 kcal/day) before the study investigation, whereas the normal to overweight individuals were not subjected to any defined diet beforehand. Medications, including statins, and/or other substances that are known to alter CYP3A4 activity/midazolam pharmacokinetics were discontinued at least seven half-lives before the investigational day, and none of the patients used any CYP3A4 inducers or time-dependent inhibitors. The study was approved by the Regional Committee for Medical and Health Research Ethics (2013/2379/REK) and complied with the Declaration of Helsinki. All patients signed a written informed consent before study participation.

### Study investigation

Blood samples for determination of plasma 4βOHC concentrations and a 24-h pharmacokinetic investigation using semi-simultaneous oral and intravenous dosing of midazolam were collected from all patients. From 10:00 p.m. before the investigation, patients abstained from food, drink (except water), and drugs. On the investigational day, patients first met for blood sampling before 1.5 mg oral midazolam syrup was administered, followed by 1.0 mg intravenous midazolam 4 h later. Blood samples for determination of midazolam plasma concentrations were collected from a peripheral venous catheter before and at 0.25, 0.5, 1, 1.5, 2, 3, 4, 4.25, 2.5, 5, 5.5, 6, 8, 10, 12, 23, and 24 h following oral dosing. The pharmacokinetic investigation has been described in more detail previously [37]. In vivo midazolam pharmacokinetic data were available in 92 patients. The day after the study investigation, paired liver and jejunum biopsies were obtained during RYGB (*n* = 38), and liver biopsies were obtained during cholecystectomy (*n* = 18), as previously described [9, 38].

Body composition was measured with Inbody 720, Body Composition Analyzer (Biospace, Korea). The NAFLD liver fat score was calculated as suggested by Kotronen et al. [39]. Standard clinical chemistry analyses were performed in fresh blood samples at the Department of Laboratory Medicine, Vestfold Hospital Trust, Tønsberg, Norway. Plasma concentrations of high-sensitivity C-reactive protein (hs-CRP) were measured using immunoturbidimetry (Advia Chemistry XPT systems, Siemens) at Fürst Medical Laboratory, Oslo, Norway. Blood samples for determination of 4βOHC and midazolam concentrations were drawn in K2-EDTA vacutainer tubes on ice and centrifuged for 10 min at 4 °C (1,800 g). Plasma was separated into Cryovials and frozen within 1 h at − 70 °C until analysis.

### Bioanalytical assays

#### 4βOHC

4βOHC plasma concentrations were determined by a previously described assay [40] with an added filtration step [41]. In short, 4βOHC was de-esterified from fatty acids by ethanolic sodium methoxide and isolated from plasma by liquid–liquid extraction with hexane. Extracts were evaporated by nitrogen and reconstituted in methanol before filtration. After methanol reconstitution, the samples were frozen at − 20 °C for 15 min, and filtered in specialized Eppendorf filter tubes (Costar Spin-x HPLC Micro Centrifuge Filter, 0,2 µM Nylon Filter) through centrifugation (2,000 g for 6 min at 2 °C). The filtered extract was used for UPLC-MS/MS analysis. An Acquity ultra-performance liquid chromatograph (UPLC) followed by a Micromass Quattro micro tandem mass spectrometry (MS) detector (Waters, Milford, MA) was used for quantitative analysis. Chromatographic separation was achieved on a BEH C18 column RP-shield (1.7 μm, 1 × 100 mm) from Waters with a mobile phase of water and methanol. After atmospheric pressure chemical ionization, detection was obtained with multiple reaction monitoring at m/z 385.25 -> 367.45 (4βOHC) and m/z 392.30 -> 374.50 (4βOHC-D7; internal standard). The lower limit of quantification (LLOQ) was 10 ng/mL. Intra‐ and interday imprecision and inaccuracy were < 15% at 10 ng/mL and < 4% at 644 ng/mL (*n* = 6) [40]. All samples were stored at − 70 °C between sampling and analysis.

#### Midazolam

The plasma concentrations of midazolam were determined by liquid–liquid sample extraction followed by a previously validated UHPLC-MS/MS method [42]. The sample preparation in short, 50 ng/mL internal standard (deuterated midazolam (MDZ-d6)), was added to a 5-mL Eppendorf tube, evaporated to dryness by nitrogen gas followed by subsequent addition of 250 µL of sample plasma. Aqueous ammonia (0.5 M) was added to the tubes in a 1:1 ratio (250 µL) and agitated prior to the addition of the extracting solvent (1.0 mL of ethyl acetate). The sample was mixed for 10 min (Invitrogen Hulamixer, Thermo Fischer, Scientific Inc) prior to being centrifuged at 2,500 g for 10 min at room temperature (Heraeus Megafuge 16R-centrifuge, Thermo Fisher Scientific Inc). The extraction was carried out twice before the organic phase was transferred to a new Eppendorf tube, evaporated to dryness under nitrogen (60 °C), and subsequently reconstituted in 50 µL 5% ACN in 0.05 M ammonium acetate buffer (pH 4.4). The sample was mixed and transferred to micro vials before UHPLC-MS/MS analysis.

The UHPLC-MS/MS method was carried out in two labs due to limited capacity. The instrument in lab 1 was a Thermo Fisher Scientific Vanquish HPLC coupled to a Thermo Fisher Accucore (C18 2.1 × 50 mm) analytical column and a Thermo Fisher Scientific Altis QqQ MS. In lab 2, a Waters (Waters, Milford, MA) Acquity UPLC with a BEH C18 RP-shield (1.7 μm, 1 × 100 mm) analytical column coupled to a Micromass Quattro micro tandem MS was used. The chromatography was carried out with a flow rate of 0.4 mL/min and 0.2 mL/min and gradient elution for lab 1 and lab 2, respectively. Mobile phase A was 5% acetonitrile in 10 mM ammonium formate, and mobile phase B was 90% acetonitrile and 10% methanol. Positive electrospray ionization and selected reaction monitoring were performed with compound-tuned conditions. For both labs, acquisition was carried out by the MRM transitions m/z 326.1 -> m/z 249.1 and m/z 326.1 -> m/z 291.2 for midazolam, and m/z 332.1 -> m/z 252.1 and m/z 332.1 -> m/z 203.1 for midazolam-d6 in positive mode.

Inter-laboratory cross-validation was performed using clinical study samples, and the bias was within ± 12.5%. Calibrators and quality control (QC) samples were prepared in blank plasma and analyzed in each analytical run. Eight calibrators in the range of 0.1 to 20 ng/mL were applied, and back-calculated values of calibrators within 80 to 120% were accepted. LLOQ was 0.1 ng/mL, and the upper limit of quantification (ULOQ) was 20 ng/mL. Samples with midazolam concentrations above ULOQ were diluted in blank plasma and reanalyzed. Dilution integrity with dilution factors of 1/2, 1/5, 1/10, 1/20, and 1/50 was established; mean accuracy ranged from 88.9 to 103.8%, and the imprecision was < 4.5%. Within-series and between-series performance were assessed with the resulting coefficient of variation < 12.3%, and the mean accuracy ranged from 99.3 to 104.3%.

### Liver and jejunum biopsies

The biopsies were transferred into individual cryotubes, snap-frozen in liquid nitrogen directly upon sampling, and stored at − 80 °C until analysis.

#### CYP3A4 protein quantification

Proteins were extracted from liver and jejunum biopsies in an SDS-containing (2% w/v) lysis buffer and quantified as previously described [43]. In short, samples were processed with the multi-enzyme digestion filter-aided sample preparation protocol, using LysC and trypsin [44]. Proteomics analysis was performed with Q Exactive HF or Q Exactive HF-X. MS data were processed with MaxQuant (version 1.6.10.43) [45], using the human UniProtKB. Spectral raw intensities were normalized with variance stabilization [46] and were subsequently used to calculate the protein concentrations using the total protein approach [47].

#### Preparation of microsomes and ex vivo CYP3A4 activity assay

Methods for quantifying CYP3A4 activity in liver and jejunum biopsies have been described previously [38]. In brief, liver and jejunum biopsies were prepared with a Potter–Elvehjem homogenizer, and differential centrifugation was used to isolate microsomal fractions. Midazolam was used as a probe drug and the formation of the metabolite 1′-hydroxymidazolam as a probe reaction for CYP3A4 activity. Activity incubation was carried out at 37 °C for 20 min, and midazolam was added in eight different concentrations. The incubation was terminated using ice-cold acetonitrile containing internal standard. Quantification of 1′-hydroxymidazolam was performed using a triple quadrupole mass spectrometer coupled to an ACQUITY UPLC I-class system (Waters XevoTM TQ-S; Waters Corporation, Milford, MA) equipped with an electrospray ionization source as previously described [38].

### Genotype analyses

*CYP3A4* and *CYP3A5* variant alleles were analyzed using Taqman-based real-time polymerase chain reaction assays implemented for routine pharmacogenetic analyses at the Center for Psychopharmacology, Diakonhjemmet Hospital, Norway. *CYP3A4*, the reduced function allele*22 (rs35599367), and *CYP3A5*, the *null* allele *3 (rs776746), were included in the analysis. All alleles were in Hardy–Weinberg equilibrium.

### Pharmacokinetic calculations

In vivo absolute bioavailability, apparent oral clearance, and systemic clearance of midazolam from the pharmacokinetic investigation were determined using a previously developed population pharmacokinetic model [37]. Briefly, the modeling was performed using the non-parametric adaptive grid approach implemented in Pmetrics (version 1.5.2) for R (version 3.6.2) [48, 49]. A catenary three-compartment model with absorption lag-time and first-order elimination from the central compartment described the data adequately. The enzyme kinetic parameters from the ex vivo CYP3A4 activity investigation were determined using untransformed data and GraphPad prism 7 by fitting the reaction velocity versus the substrate concentration data to the Michaelis–Menten model or the substrate inhibition model, as previously described [38]. The Michaelis constant (*K*_m_) values were adjusted for the fraction of unbound drug in microsomes, which was predicted from the physicochemical properties of the substrates and microsomal protein concentration using the Simcyp prediction tool (https://members.simcyp.com/account/tools/fumic). The unbound CL_int_ (CL_int,u_) was calculated from the ratio of maximum velocity (*V*_max_) to the unbound *K*_m_. Due to limited capacity, the hepatic and jejunum microsomal CL_int,u_ were determined in 20 RYGB patients.

### Data and statistical analysis

Neither 4βOHC nor most of the other variables passed the normality tests (including the Shapiro–Wilk test and visual inspection of plots), and non-parametric statistical tests were therefore used in the statistical analyses. Spearman’s rank-order correlation test was used to describe the rank-based measure of association. Wilcoxon rank-sum test was used to compare patients with obesity with normal to overweight individuals. A *p* value < 0.05 was considered statically significant. Data are presented as median difference (95% confidence interval (CI)) if not otherwise stated. Demographic data are presented as mean ± standard deviation (SD). All statistical analyses were performed using R for Windows (version 4.1.2) [49].

## Results

### Patient characteristics

Patient characteristics at the investigational day are given in Table 1. The patients had a mean age of 46 ± 11 years, 70% were women, and 98% were Caucasian. The majority (94%) were genotypic normal CYP3A4 metabolizers (CYP3A4 *1/*1), and 10% also expressed functional CYP3A5 (CYP3A5 *1/*3). In line with the inclusion criteria, mean BMI was higher in patients with obesity compared with normal to overweight individuals. Patients with obesity had lower serum total cholesterol compared with the normal to overweight individuals (− 0.60 mmol/L [95% CI: − 1.0, − 0.00]) but higher NAFLD liver fat score (2.8 [95% CI: 1.9, 3.6]) and hs-CRP (2.1 mg/L [95% CI: 1.1, 3.9]). Twenty percent of the patients received treatment with cholesterol-lowering drugs (mainly statins) (patients with obesity = 19, normal to overweight = 0).

**Table 1:** Patient characteristics at the study investigation. Data are described as mean ± SD or absolute numbers (%)

	**Patients with obesity** ***n* = 78**	**Normal to overweight individuals** ***n* = 18**
Age (years)	47 ± 10	42 ± 15
Sex (male/female)	26/52	3/15
Body weight (kg)	121 ± 22	71 ± 11
BMI (kg/m^2^)	41 ± 5.8	25 ± 3.5
Total cholesterol (mmol/L)	3.9 ± 0.95	4.4 ± 0.87
Triglycerides (mmol/L)	1.3 ± 0.59	0.93 ± 0.47
LDL (mmol/L)	2.4 ± 0.82	2.5 ± 0.77
HDL (mmol(L)	0.96 ± 0.20	1.5 ± 0.36
hs-CRP (mg/L)	5.7 ± 6.0	2.5 ± 3.8
ALT (U/L)	39 ± 21	22 ± 15
NAFLD liver fat score	1.1 ± 2.0	− 1.7 ± 1.1
CYP3A4 genotype (likely phenotype)		
*1/*1 (normal metabolizer)	74 (95%)	16 (89%)
*1/*22 (intermediate metabolizer)	4 (5%)	2 (11%)
CYP3A5 genotype (likely phenotype)		
*1/*3 (intermediate metabolizer)	7 (9%)	3 (17%)
*3/*3 (poor metabolizer)	71 (91%)	15 (83%)

*ALT* alanine aminotransferase, *BMI* body mass index, *CYP* cytochrome P450, *HDL* high-density lipoproteins, *hs-CRP* high-sensitivity C-reactive protein, *LDL* low-density lipoproteins, *NAFLD* non-alcoholic fatty liver disease

### Correlations between 4βOHC and CYP3A4 metrics

**Fig. 1:**
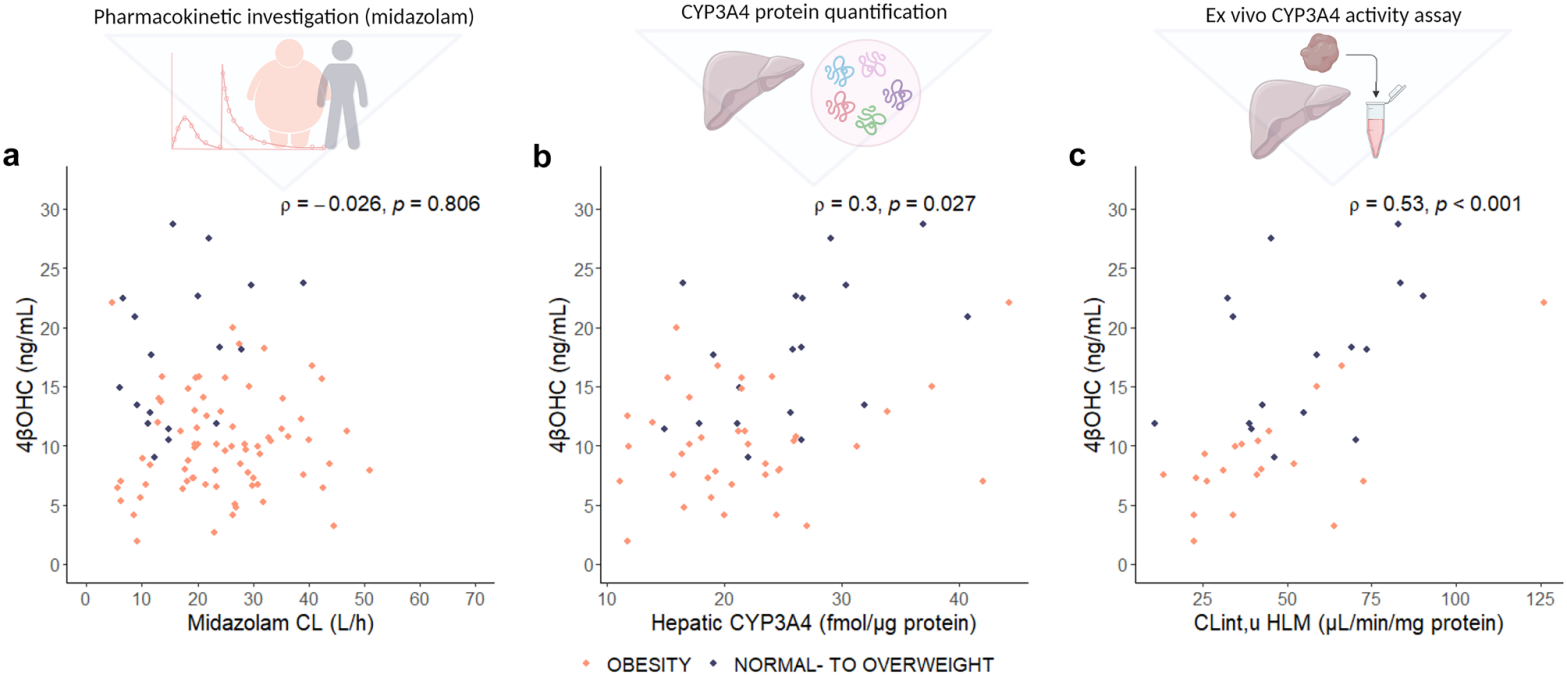
Hepatic CYP3A4 metrics. Correlation between (**a**) systemic midazolam clearance (CL) and 4βOHC concentrations^a^, (**b**) hepatic CYP3A4 concentration^b^ and 4βOHC concentrations, and (**c**) clearance intrinsic for midazolam 1’-hydroxylation in human liver microsomes^c^ and 4βOHC concentrations. Spearman’s rho (*ρ*) is the correlation coefficient, and the *p* value is from the Spearman rank correlation analysis. ^a^Available in 92 patients (patients with obesity = 74, normal to overweight individiuals = 18). ^b^Available in 56 patients (patients with obesity = 38, normal to overweight individuals = 18). ^c^Available in 36 patients (RYGB = 20, normal to overweight individuals = 16). *Abbreviations: CL*_*int,u,*_* clearance intrinsic unbound; CYP, cytochrome P450; HLM, human liver microsomes; 4βOHC, 4-beta hydroxycholesterol*

**Fig. 2:**
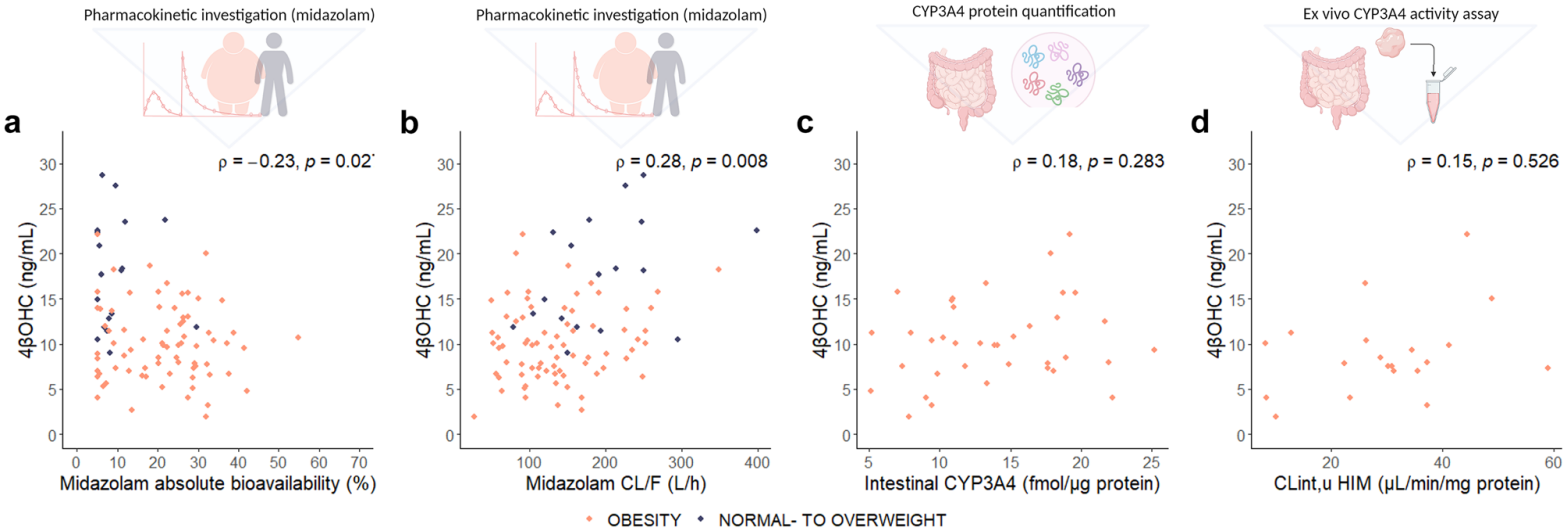
Intestinal CYP3A4 metrics. Correlation between (**a**) midazolam absolute bioavailability^a^ and 4βOHC concentrations, (**b**) apparent oral midazolam clearance (CL/F)^a^ and 4βOHC concentrations, (**c**) jejunum CYP3A4 concentration^b^ and 4βOHC concentrations, and (**d**) clearance intrinsic for midazolam 1′-hydroxylation in human intestinal microsomes^c^ and 4βOHC concentrations. Spearman’s rho (*ρ*) is the correlation coefficient, and the *p* value is from the Spearman rank correlation analysis. ^a^Available in 92 patients (patients with obesity = 74, normal to overweight individuals = 18). ^b^Available in 37 RYGB-patients. ^c^Available in 20 RYGB-patients. *Abbreviations: CL*_*int,u,*_* clearance intrinsic unbound; CYP, cytochrome P450; HIM, human intestinal microsomes; 4βOHC, 4-beta hydroxycholesterol*

4βOHC concentrations were positively correlated with both hepatic CYP3A4 concentrations (*ρ* = 0.30, *p* = 0.027) and hepatic microsomal CL_int,u_ (*ρ* = 0.53, *p* < 0.001), but not with systemic midazolam clearance (*ρ* =  − 0.03, p = 0.81) (Fig. 1). The lack of correlation between 4βOHC concentrations and systemic midazolam clearance was also shown when the patients with obesity and the normal to overweight individuals were analyzed separately (Supplementary Fig. S1). Hepatic microsomal activity was significantly correlated with hepatic CYP3A4 concentrations (*ρ* = 0.51, *p* = 0.001), but no correlation was observed between systemic midazolam clearance and hepatic microsomal CL_int,u_ (Supplementary Fig. S2). 4βOHC concentrations were not significantly correlated with intestinal CYP3A4 concentrations or intestinal microsomal CL_int,u_ (Fig. 2c and d). However, 4βOHC plasma concentrations showed an inverse correlation with absolute bioavailability of midazolam (*ρ* =  − 0.23, *p* = 0.027), and a positive correlation with apparent oral clearance (*ρ* = 0.28, *p* = 0.008) (Fig. 2a and b). Absolute values of the different CYP3A metrics are reported in Supplementary Table S1.

In the patients with obesity, neither 4βOHC concentrations, systemic midazolam clearance, nor absolute bioavailability changed during the 3-week low-energy diet period (Supplementary Fig. S3). However, due to the low-fat low-energy diet and weight loss, the total cholesterol was lowered in this period. All correlations were thus repeated using the 4βOHC/cholesterol ratio (4βOHC/C). The results from these analyses showed a similar pattern (Supplementary Figs. S4 and S5).

### Impact of genotype and patient characteristics on 4βOHC

**Fig. 3:**
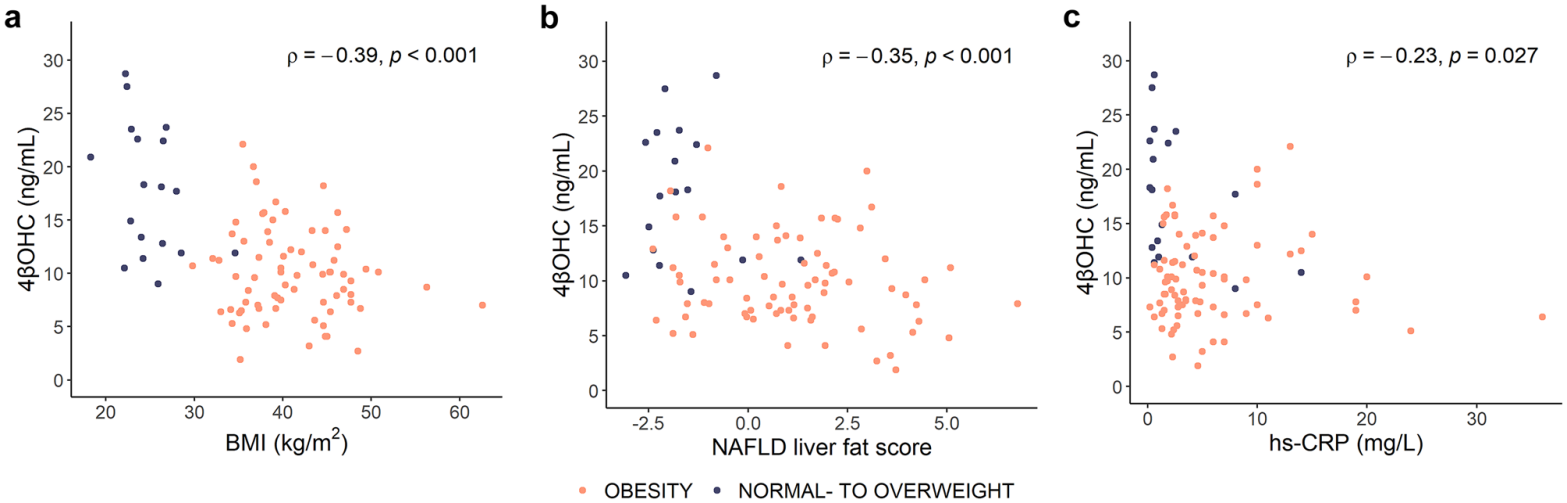
Clinical variables and 4βOHC. Correlation between (**a**) BMI and 4βOHC concentrations (*n* = 96), (**b**) NAFLD liver fat score and 4βOHC concentrations (*n* = 95), and (**c**) hs-CRP and 4βOHC concentrations (*n* = 96). Spearman’s rho (*ρ*) is the correlation coefficient, and the *p* value is from the Spearman rank correlation analysis. *Abbreviations: BMI, body mass index; hs-CRP, high-sensitivity C-reactive protein; NAFLD, non-alcoholic fatty liver disease; 4βOHC, 4-beta hydroxycholesterol*

An analysis of 4βOHC concentrations based on genotype did not indicate elevated 4βOHC in the individuals expressing CYP3A5 *1/*3 compared with CYP3A5 *3/*3 expressors, neither in normal to overweight individuals (*p* = 0.95) nor in patients with obesity (*p* = 0.33) (Supplementary Fig. 6a and b). However, 4βOHC concentrations were lower in patients with obesity expressing CYP3A4 *1/*22 compared with CYP3A4 *1/*1 (*p* = 0.024), whereas in the normal to overweight individuals, the difference did not reach statistical significance (*p* = 0.12) (Supplementary Fig. S6c and d). BMI was inversely correlated with 4βOHC concentrations: *ρ* =  − 0.39 (*p* < 0.001) (Fig. 3a), and median 4βOHC was 1.9-fold higher in normal to overweight individuals compared with patients with obesity (median difference 7.4 ng/mL [95% CI: 4.4, 11]) (Supplementary Fig. S7a). Both NAFLD liver fat score and hs-CRP were inversely correlated with 4βOHC: *ρ* =  − 0.35 (*p* < 0.001) and *ρ* =  − 0.23 (*p* = 0.027), respectively (Fig. 3b and c). Absolute bioavailability and systemic clearance of midazolam were 2.8-fold and 1.6-fold higher in the patients with obesity compared with the normal to overweight individuals (13% [95% CI: 5.6, 18] and 7.7 L/h [95% CI: 2.3, 13], respectively) (Supplementary Fig. S7b and c). There was no sex difference in 4βOHC concentrations in the study population (median difference between females and males: 1.8 ng/mL [95% CI: − 0.30, 3.9]).

## Discussion

In this study, a comprehensive investigation of 4βOHC as an endogenous biomarker for CYP3A4 activity was performed. To our knowledge, this is the first study where 4βOHC has been compared with three other CYP3A4 metrics: intestinal and hepatic microsomal CYP3A4 activity and protein expression in paired liver and jejunum samples, as well as absolute bioavailability, apparent oral clearance, and systemic clearance of midazolam in vivo. The main findings were that 4βOHC concentrations were positively correlated with hepatic microsomal CYP3A4 activity and hepatic CYP3A4 expression. Equally important, this study also provides evidence of a negligible contribution from intestinal CYP3A4 to the formation of 4βOHC.

The moderate correlation between 4βOHC concentrations and hepatic microsomal activity (CL_int,u_) supports that 4βOHC concentrations reflect individual CYP3A4 metabolic capacity and provides evidence that 4βOHC is a suitable marker for hepatic CYP3A4 phenotype. However, there was no correlation between 4βOHC concentrations and systemic clearance of midazolam, the gold standard method to assess CYP3A4 activity. The correlation between these two metrics has been investigated in multiple studies previously, with conflicting findings varying from no correlation as in this study to a weak or moderate correlation [24–27, 50, 51]. Even though systemic clearance of midazolam currently is the preferred method for CYP3A4 phenotyping, it may have some important limitations in special populations, e.g., patients with obesity, as shown in the present study. Surprisingly, we observed no correlation between hepatic microsomal activity (CL_int,u_) and in vivo systemic midazolam clearance, suggesting that systemic midazolam clearance does not solely reflect CYP3A4 activity in the present population. In contrast to hepatic microsomal CL_int,u_, in vivo systemic midazolam clearance may also be influenced by differences in hepatic blood flow given its medium to high extraction ratio [17–19] which may be especially relevant in patients with chronic diseases or in case of simultaneous physiological alterations. We have previously hypothesized that hepatic blood flow may have a more pronounced impact on systemic midazolam clearance than CYP3A4 activity in patients with obesity due to higher liver blood flow in this patient population compared with normal-weight individuals [20]. Brill et al. have also suggested that blood flow alterations seem to influence midazolam pharmacokinetics in patients with obesity [21]. This may at least partly explain why patients with obesity had lower 4βOHC concentrations in combination with higher systemic midazolam clearance compared with normal to overweight individuals, and also the lack of correlation between 4βOHC and systemic midazolam clearance in the present study. 4βOHC may therefore be an especially relevant biomarker to include in midazolam-based CYP3A4 phenotyping studies in specific patient populations where physiological factors such as altered blood flow may challenge the interpretation of the results. Additionally, there are some methodological considerations with the ex vivo CYP3A activity assay that should be taken into consideration. Microsomal activity in small liver tissue samples may not be representative of the CYP3A4 activity across the whole liver [52], the unbound fraction was estimated, and it was not accounted for variability in microsomal protein per gram liver and microsomal recovery.

Information regarding the role of intestinal CYP3A4 in 4βOHC formation has been lacking [32, 53], and uncertainty about the intestinal contribution seems to be an important limitation with 4βOHC phenotyping. We showed that neither intestinal CYP3A4 expression nor intestinal microsomal CYP3A4 activity (CL_int,u_) was correlated with 4βOHC concentrations. Even though there was a weak, but significant correlation between 4βOHC concentrations and (1) midazolam absolute bioavailability, and (2) apparent oral clearance, but no correlation with systemic clearance, these results indicate that intestinal CYP3A4 has a negligible role in the formation of 4βOHC. There have been some indications of a possible contribution from intestinal CYP3A4 in the formation of 4βOHC [40, 54]. However, a more recent study indicates that intestinal CYP3A4 is unlikely to be of significant importance for the 4βOHC formation [55], which is in line with our findings. Hence, it seems that 4βOHC is mainly appropriate for hepatic CYP3A4 phenotyping, but the relatively small sample size in our study should be kept in mind when interpreting these findings.

In this study, we also report a negative correlation between BMI and 4βOHC, and that patients with obesity have significantly lower 4βOHC concentrations compared with normal to overweight individuals. Previous studies have also found an inverse correlation between BMI and 4βOHC/C, and body weight and 4βOHC/C [10, 25]. Intestinal and hepatic CYP3A4 expression as well as CL_int,u_ using midazolam as a probe drug are also reported to be negatively correlated with BMI [8, 9]. This supports that patients with obesity have a lower CYP3A4 activity compared with normal weight individuals [7]. The normal to overweight individuals in this study had similar values of 4βOHC compared to reported values for corresponding Caucasians in other studies [10, 23, 51, 56]. Lower levels of 4βOHC were also observed by Gravel et al. in patients with a mean BMI of ~ 29 kg/m^2^, although the authors suggested that this was due to type 2 diabetes, not obesity [51], and by Woolsey et al. in patients with a mean BMI of ~ 33 kg/m^2^ and NAFLD [5]. CYP3A4 activity seems to be suppressed during inflammation [57, 58], and in agreement with this, we also found a weak inverse correlation between 4βOHC concentrations and the inflammation marker hs-CRP. Björkhem-Bergman et al. also reported a similar correlation in a study with patients with increased susceptibility to respiratory infections [59]. Furthermore, and in agreement with a previous study in patients with NAFLD and obesity [5], we observed a weak inverse correlation between NAFLD liver fat score and 4βOHC concentrations, suggesting decreased CYP3A4 activity in patients with NAFLD.

Most of the cholesterol formation to 4βOHC is accounted for by CYP3A4 metabolism, whereas the contribution from CYP3A5 is more ambiguous [12, 22, 23, 60, 61]. Hole et al. found no significant effect of CYP3A5 genotype on 4βOHC concentrations in a similar study population as ours and suggested that CYP3A5 has a limited role in the formation of 4βOHC in vivo [12]. In agreement with this, we did not observe any significant difference in 4βOHC concentrations between individuals expressing functional (CYP3A5*1/*3) and non-functional CYP3A5 (CYP3A5*3/*3). Nevertheless, we do not believe that the heterozygote CYP3A5*1 carriers in our study population (~ 10%) have impacted the interpretation of the results in this study to any significant degree. This is supported by the fact that there were no statistically significant differences between the heterozygote CYP3A5*1 carriers and the homozygote CYP3A5*3 carriers in any of the other metrics investigated. Also, there was a tendency of lower 4βOHC concentrations in CYP3A4*22 carriers (reduced function), but the interpretation is challenged by the low number of individuals carrying this allele. Overall, these results are in line with previous studies [12, 25, 50] and support that 4βOHC primarily seems to be a biomarker for CYP3A4 phenotype.

The major strength of this study includes the comprehensive investigation of three different CYP3A4 metrics in relation to 4βOHC, all obtained from the same individuals at the same time point. The patients with obesity were subjected to a 3-week low-energy diet before the study investigation, which may have influenced the results. However, as neither 4βOHC concentrations, absolute bioavailability, nor systemic clearance of midazolam changed from before to after the diet, a change in the CYP3A4 phenotype during this short period seems unlikely. Nevertheless, in addition to the fact that 20% of the patients used cholesterol-lowering drugs, cholesterol levels also decreased following the diet, which led to a significant increase in 4βOHC/C in the patients with obesity. Several studies report the 4βOHC/C ratio to account for abnormal or changing cholesterol levels. However, the concentration of 4βOHC is less than 0.002% of total cholesterol [53]. We decided to report 4βOHC concentrations, as most individuals had normal cholesterol levels, but analyses using 4βOHC/C yielded similar results. Also, Diczfalusy et al. have previously shown that variability in cholesterol levels only explains 9% of the variability in 4βOHC concentrations [23]. Another limitation includes not adjusting for multiple testing, which may increase the likelihood of type 1 errors. Additionally, the results in this study may not be applicable in other populations, as the majority of the study population were patients with obesity.

In summary, this comprehensive study showed that 4βOHC plasma concentrations reflect hepatic, but not intestinal CYP3A4 activity well. Hence, 4βOHC may be a valuable biomarker for individualized dosing of intravenously administered CYP3A4 substrate drugs. Additionally, 4βOHC is a valuable supplement to traditional phenotyping with probe drugs such as midazolam given its easy implementation and complementary information when other factors, such as disease state or simultaneous physiological alterations, are expected to influence the pharmacokinetics of CYP3A4 probe drugs.

## Supplementary information

Below is the link to the electronic supplementary material.

Supplementary file1 (DOCX 1062 KB)

## Data Availability

Access to data collected from this study, including anonymized individual-participant data, may potentially be made available following publication upon e-mail request to the corresponding author. After approval of a proposal, data will be shared with investigators whose proposed use of the data has been approved by the COCKTAIL steering committee, according to the consent given by the participants and Norwegian laws and legislations.
