# Supplementary material for: Correlations between 4β-hydroxycholesterol and hepatic and intestinal CYP3A4: protein expression, microsomal ex vivo activity, and in vivo activity in patients with a wide body weight range
Source: Eur J Clin Pharmacol. 2022 Jun 1;78(8):1289–99. doi: 10.1007/s00228-022-03336-9 (PMC9283167; doi:10.1007/s00228-022-03336-9)
Supplement: Supplementary file 1 — Supplementary file1 (DOCX 1062 KB) [file 228_2022_3336_MOESM1_ESM.docx]

**Supplementary Information**

**Correlations between 4β-hydroxycholesterol and hepatic and intestinal CYP3A4; protein expression, microsomal *ex vivo* activity, and *in vivo* activity in patients with a wide body weight range**

*European Journal of Clinical Pharmacology*

Kine Eide Kvitne^1^, Kristine Hole^2,3^, Veronica Krogstad^1^, Birgit Malene Wollmann^2^, Christine Wegler^4,5^, Line K. Johnson^6^, Jens K. Hertel^6^, Per Artursson^7^, Cecilia Karlsson^8,9^, Shalini Andersson^10^, Tommy B. Andersson^5^, Rune Sandbu^6,11^, Jøran Hjelmesæth^6,12^ Eva Skovlund^13^, Hege Christensen^1^, Rasmus Jansson-Löfmark^5^, Anders Åsberg^1,14^, Espen Molden^1,2^, Ida Robertsen^1^

**Affiliations:**

*^1^Section for Pharmacology and Pharmaceutical Biosciences, Department of Pharmacy, University of Oslo, Norway
^2^Center for Psychopharmacology, Diakonhjemmet Hospital, Oslo, Norway. ^3^Department of Life Sciences and Health, Oslo Metropolitan University, Oslo, Norway
^4^Department of Pharmacy, Uppsala University, Sweden
^5^DMPK, Research and Early Development, Cardiovascular, Renal and Metabolism (CVRM), BioPharmaceuticals R&D, AstraZeneca, Gothenburg, Sweden
^6^The Morbid Obesity Center, Vestfold Hospital Trust, Tønsberg, Norway ^7^Department of Pharmacy and Science for Life Laboratory, Uppsala University, Sweden
^8^Clinical Metabolism, Late-stage Development, Cardiovascular, Renal and Metabolism (CVRM), BioPharmaceuticals R&D, AstraZeneca, Gothenburg, Sweden
 ^9^Department of Molecular and Clinical Medicine, Institute of Medicine, Sahlgrenska Academy, University of Gothenburg, Gothenburg, Sweden
^10^Oligonucleotide Discovery, Discovery Sciences, R&D, AstraZeneca, Gothenburg, Sweden
^11^Deparment of Surgery, Vestfold Hospital Trust, Tønsberg, Norway ^12^Department of Endocrinology, Morbid Obesity and Preventive Medicine, Institute of Clinical Medicine, University of Oslo, Norway
^12^Department of Public Health and Nursing, Norwegian University of Science and Technology, NTNU, Trondheim, Norway
^13^Department of Transplant Medicine, Oslo University Hospital, Oslo, Norway*

**Corresponding author:**

Kine Eide Kvitne
Department of Pharmacy, University of Oslo, P.O. Box 1068 Blindern, 0316 Oslo, Norway
E-mail: [k.e.kvitne@farmasi.uio.no](mailto:k.e.kvitne@farmasi.uio.no)

**
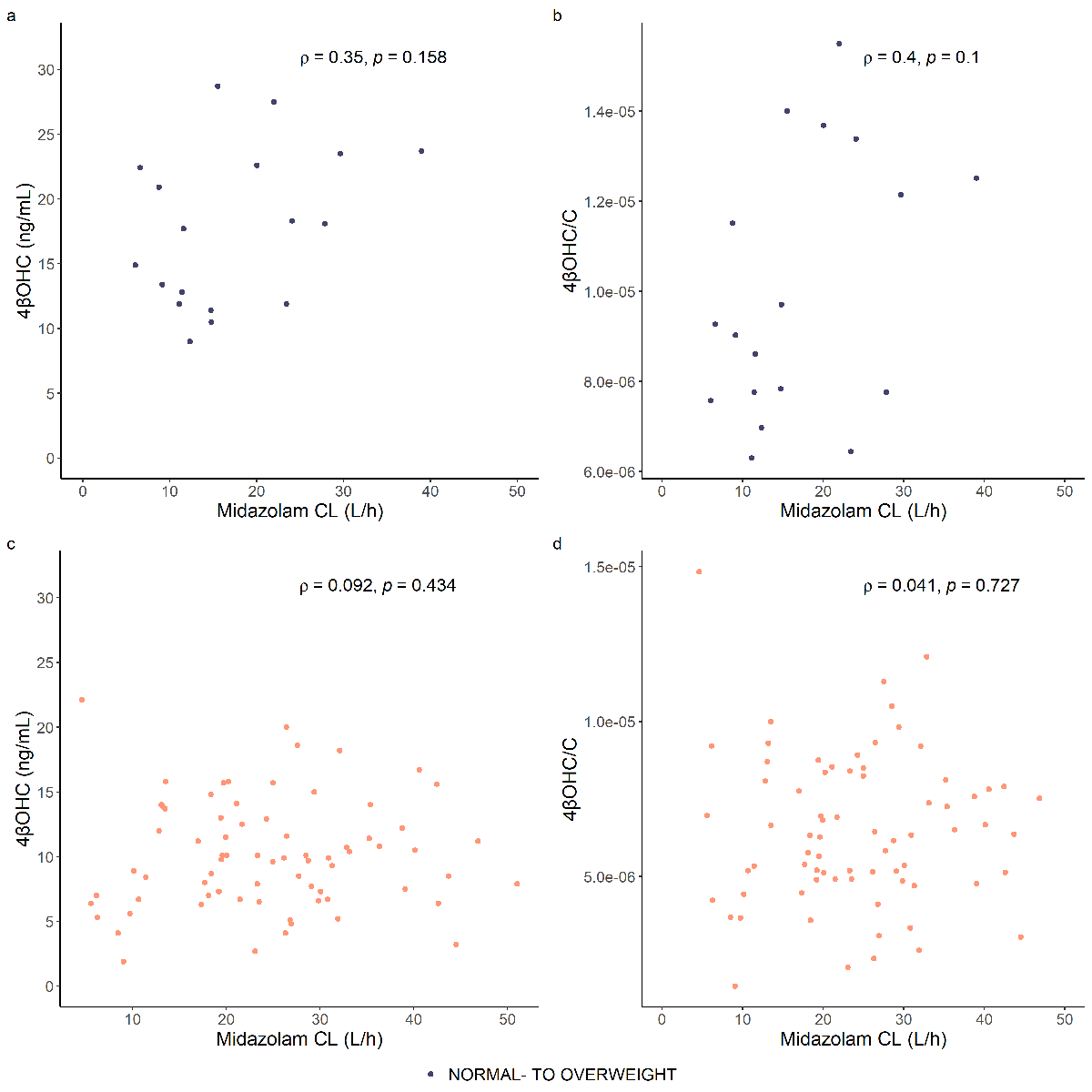
**

**Fig S1. Association between systemic midazolam clearance and (a) 4βOHC concentrations, and (b) 4βOHC/C in normal- to overweight individuals (n=18). Association between systemic midazolam clearance and (c) 4βOHC concentrations, and (d) 4βOHC/C in patients with obesity (n=78).** Spearman’s rho (ρ) is the correlation coefficient, and the p value is from the Spearman rank correlation analysis

*Abbreviations: 4βOHC, 4-beta hydroxycholesterol; 4βOHC/C, 4-beta hydroxycholesterol/cholesterol ratio*


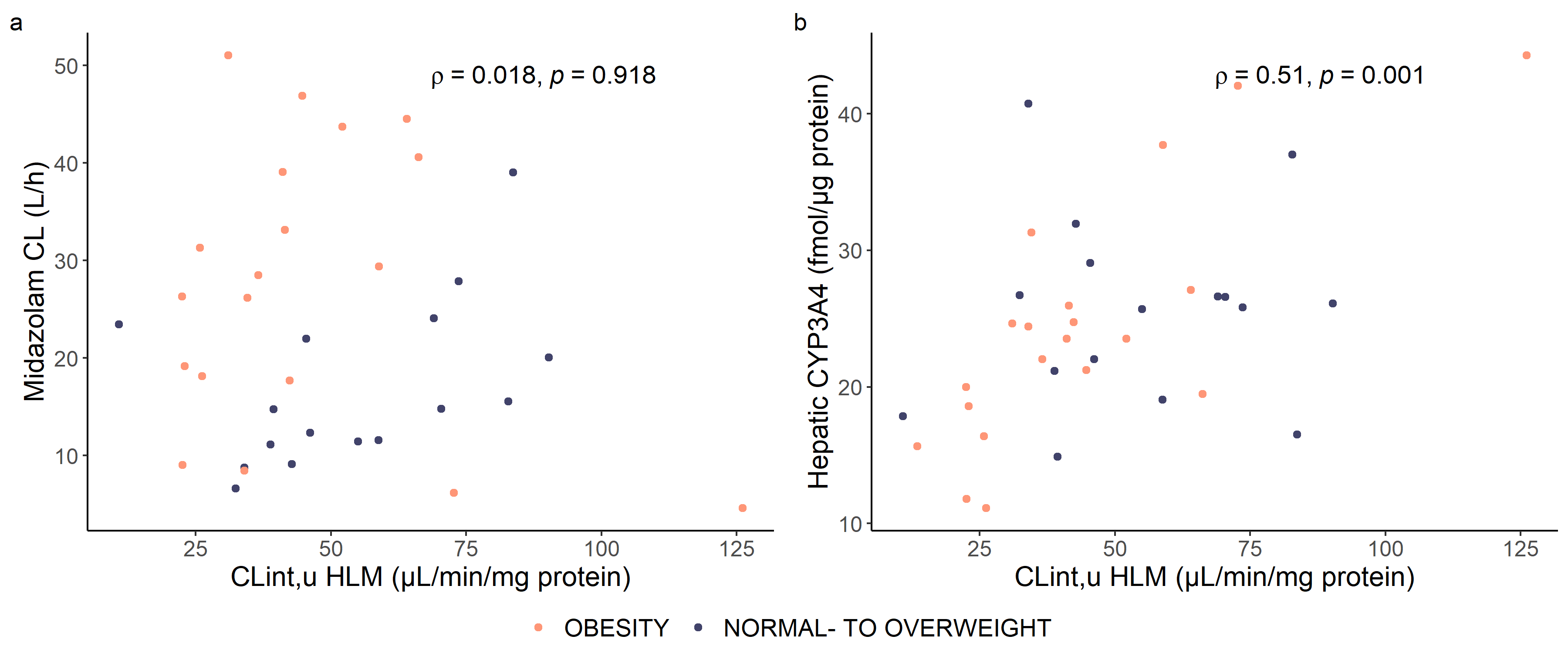
 **Fig. S2. Association between clearance intrinsic for midazolam 1’-hydroxylation in human liver microsomes and (a) systemic midazolam clearance (n=36), and (b) hepatic CYP3A4 expression (n=36).** Spearman’s rho (ρ) is the correlation coefficient, and the p value is from the Spearman rank correlation analysis

*Abbreviations: CL_int,u,_ clearance intrinsic, unbound; CYP, cytochrome P450; HLM, human liver microsomes*

**Table S1. CYP3A metrics in the two study groups.** Data are presented as median [IQR].

|  | **Patients with obesity**  n=78 | **Normal- to overweight individuals**  n=18 |
| --- | --- | --- |
| Absolute bioavailability (%) ^a^ | 22  [12, 29] | 7.8  [5.7, 11] |
| Systemic clearance (L/h) ^a^ | 24  [18, 31] | 15  [11, 23] |
| Apparent oral clearance (L/h) ^a^ | 134  [93, 173] | 185  [145, 243] |
| 4βOHC (ng/mL) | 9.7  [7.1, 12] | 18  [12, 23] |
| Hepatic CYP3A4 (fmol/µg protein)  ^b^ | 21  [17, 25] | 26  [21, 28] |
| CL_int,u_ HLM (µL/min/mg protein)  ^c^ | 39  [26, 54] | 51  [39, 71] |
| Intestinal CYP3A4 (fmol/µg protein) ^d^ | 13  [9.8, 18] | *NA* |
| CL_int,u_ HIM (µL/min/mg protein) ^e^ | 31  [23, 37] | *NA* |

*Abbreviations: CL_int,u,_ clearance intrinsic unbound; CYP, cytochrome P450; HIM, human intestinal microsomes; HLM, human liver microsomes; 4βOHC, 4β-hydroxycholesterol*
*^a^* Midazolam population pharmacokinetic model derived parameters
*^b^* Only available in 56 individuals (obesity).
*^c^* Only available in 36 individuals (obesity).
*^d^* Only available in 37 individuals (obesity).
*^e^* Only available in 20 individuals (obesity).


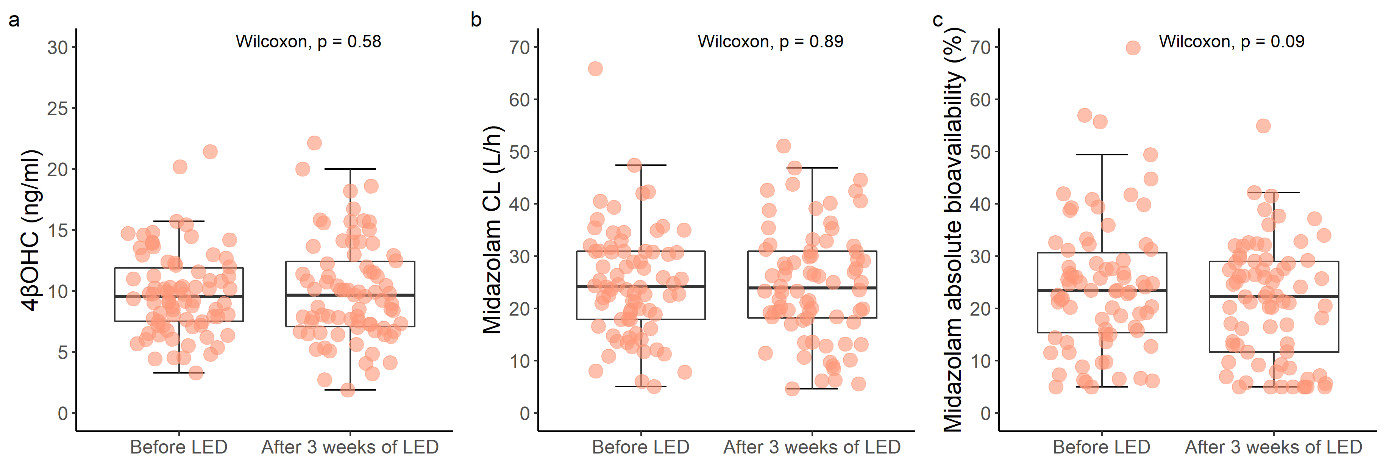
**Fig. S3. CYP3A4 metrics before and after three weeks of LED.** Boxplot with individual points of (a) 4βOHC concentrations (n=96), (b) systemic midazolam clearance (n=92), and (c) midazolam absolute bioavailability (n=92) in patients with severe obesity. Wilcoxon signed-rank test was used to compare change from week 0 to week 3

*Abbreviations: 4βOHC, 4-beta hydroxycholesterol*


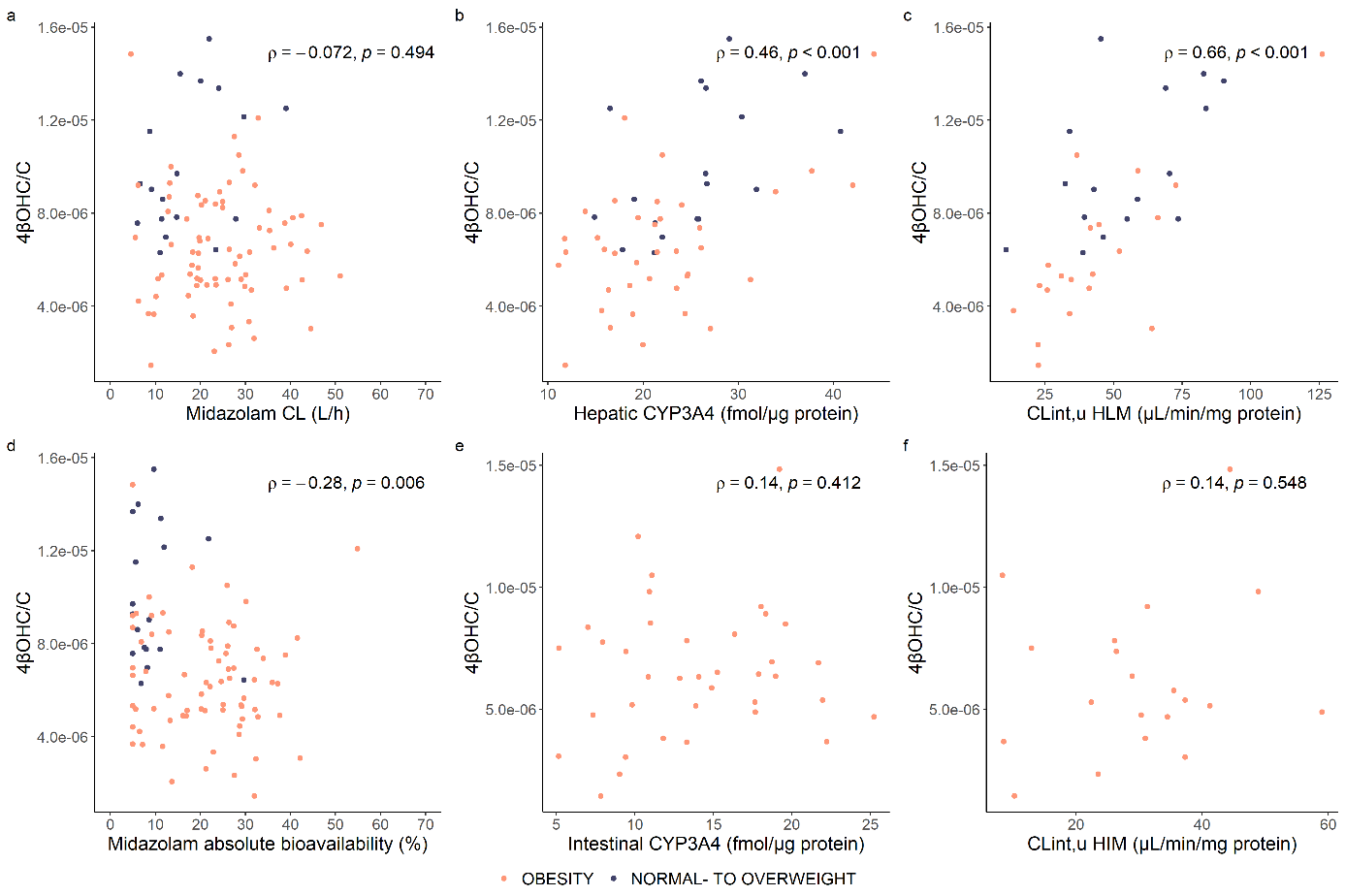
**Fig. S4. CYP3A4 metrics and 4βOHC/C.** Association between (a) systemic midazolam clearance and 4βOHC/C (n=92), (b) hepatic CYP3A4 expression and 4βOHC/C (n=56), (c) clearance intrinsic for midazolam 1’-hydroxylation in human liver microsomes and 4βOHC/C (n=36), (d) midazolam absolute bioavailability and 4βOHC/C (n=92), (e) jejunum CYP3A4 expression and 4βOHC/C (n=37), and (f) clearance intrinsic for midazolam 1’-hydroxylation in human intestinal microsomes and 4βOHC/C (20). Spearman’s rho (ρ) is the correlation coefficient, and the p value is from the Spearman rank correlation analysis.

*Abbreviations: CL_int,u,_ clearance intrinsic, unbound; CYP, cytochrome P450; HIM, human intestinal microsomes; HLM, human liver microsomes; 4βOHC/C, 4-beta hydroxycholesterol/cholesterol ratio*

**
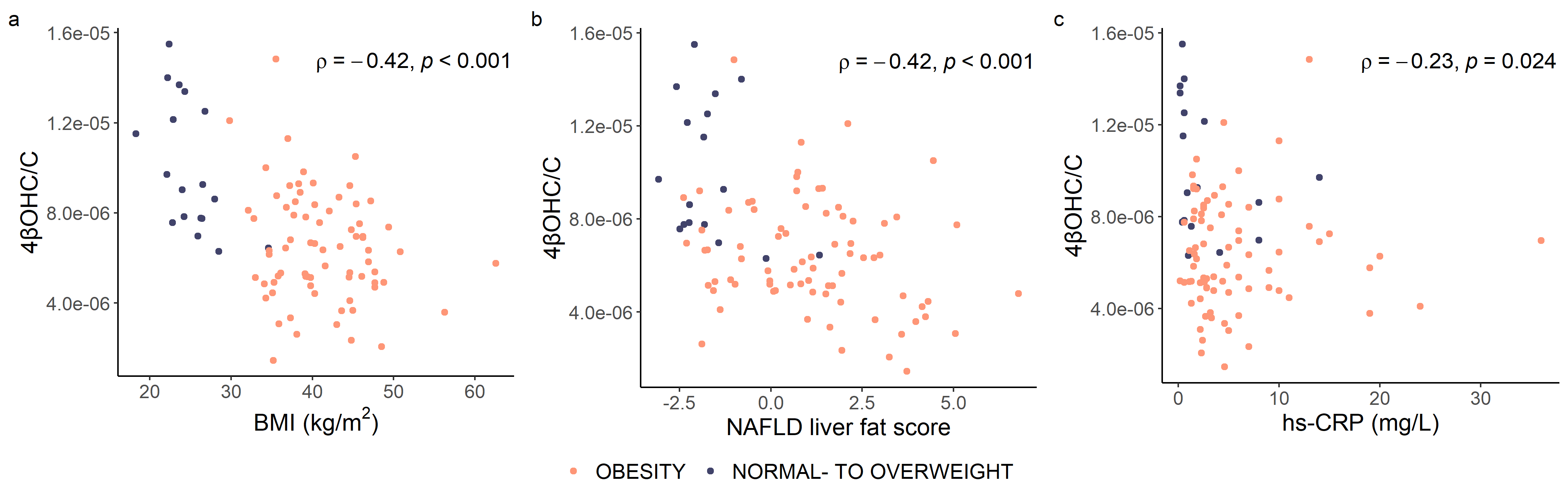
Fig. S5. Clinical variables and 4βOHC/C**. Association between (a) BMI and 4βOHC/C (n=96), (b) NAFLD liver fat score and 4βOHC/C (n=95), and (c) high-sensitivity C-reactive protein and 4βOHC/C (n=96). Spearman’s rho (ρ) is the correlation coefficient, and the p value is from the Spearman rank correlation analysis

*Abbreviations: BMI, body mass index; hs-CRP, high-sensitivity C-reactive protein; NAFLD, non-alcoholic fatty liver disease; 4βOHC/C, 4-beta hydroxycholesterol/cholesterol ratio*

**
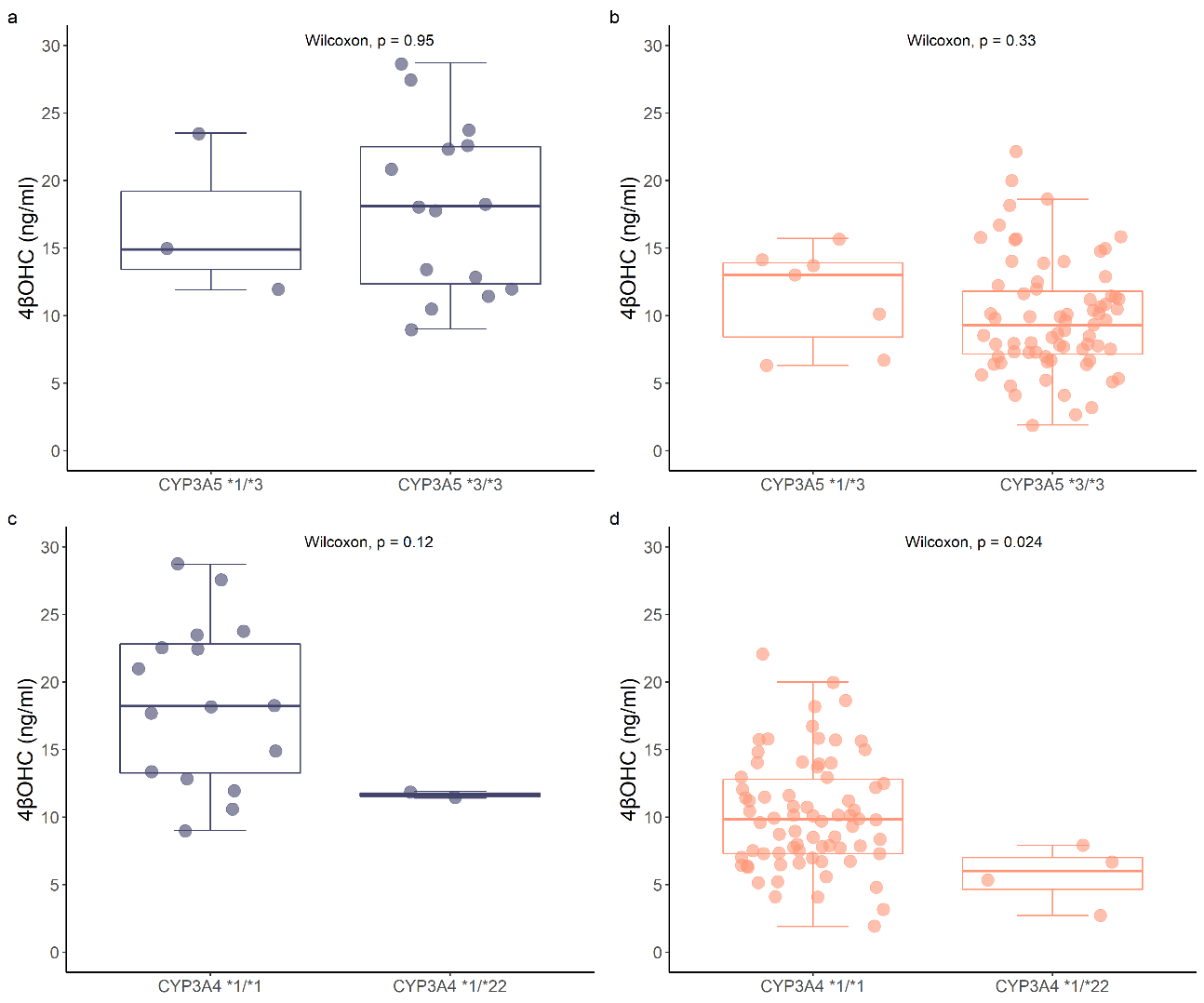

Fig. S6. 4βOHC and genotype.** 4βOHC concentrations in (a) normal- to overweight individuals with *CYP3A5 *1/*3* (n=3) or *CYP3A5 *3/*3* (n=15) genotype, (b) patients with obesity with *CYP3A5 *1/*3* (n=7) or *CYP3A5 *3/*3* (n=71) genotype, (c) normal- to overweight individuals with *CYP3A4 *1/*1* (n=16) and *CYP3A4 *1/*22* (n=2), and (d) patients with obesity *CYP3A4 *1/*1* (n=74) and *CYP3A4 *1/*22* (n=4). Wilcoxon rank-sum test was used to compare the two groups

*Abbreviations: CYP, cytochrome P450; 4βOHC, 4-beta hydroxycholesterol*

**
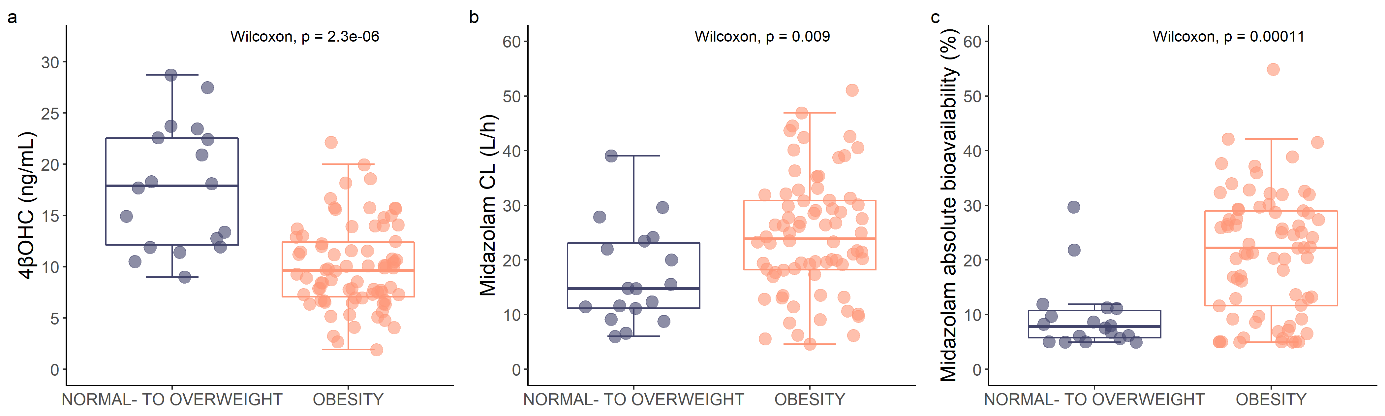
Fig. S7. CYP3A4 metrics in patients with obesity and normal- to overweight individuals.** Boxplot with individual points of (a) 4βOHC concentrations (n=96), (b) systemic midazolam clearance (n=92), and (c) midazolam absolute bioavailability (n=92). Wilcoxon rank-sum test was used to compare the two groups

*Abbreviations: 4βOHC, 4-beta hydroxycholesterol*
